# Case report: L5 tomita En bloc spondylectomy for oligometastatic liposarcoma with post adjuvant stereotactic ablative radiotherapy

**DOI:** 10.3389/fsurg.2023.1110580

**Published:** 2023-03-09

**Authors:** Priyanshu Saha, Mohsen Raza, Angelo Fragkakis, Bisola Ajayi, Timothy Bishop, Jason Bernard, Aisha Miah, Shane H. Zaidi, Mohamed Abdelhamid, Pawan Minhas, Darren F. Lui

**Affiliations:** ^1^ School of Medicine, St George's, University of London, United Kingdom; ^2^ Department of Complex Neurosurgery, St George's University Hospitals NHS Foundation Trust, United Kingdom; ^3^ Department of Sarcoma, Royal Marsden NHS Foundation Trust, United Kingdom; ^4^ Department of Vascular Surgery, St George's University Hospitals NHS Foundation Trust, United Kingdom

**Keywords:** spinal oligometastatic disease, sarcoma, case report, dual stage tomita *en bloc* spondylectomy, carbon fibre (CF), spine surgery, stereotactic ablative radiotherapy (SABR), oncological spine surgery

## Abstract

**Introduction:**

Tomita En-bloc spondylectomy of L5 is one of the most challenging techniques in radical oncological spine surgery. A 42-year-old female was referred with lower back pain and L5 radiculopathy with a background of right shoulder liposarcoma excision. CT-PET confirmed a solitary L5 oligometastasis. MRI showed thecal sac indentation hence wasn't suitable for Stereotactic Ablative Radiotherapy (SABR) alone. The seeding nature of sarcoma prevents the indication of separation surgery hence excisional surgery is considered for radical curative treatment. This case report demonstrates dual-staged modified TES including the utilisation of novel techniques to allow for maximum radical oncological control in the era of SABR and lesser invasive surgery.

**Methods:**

First-stage: Carbonfibre pedicle screws planned from L2 to S2AI-Pelvis, aligned, to her patient-specific rods. Radiofrequency ablation of L5 pedicles prior to osteotomy was performed to prevent sarcoma cell seeding. Microscope-assisted thecal sac tumour separation and L5 nerve root dissection was performed. Novel surgical navigation of the ultrasonic bone-cutter assisted inferior L4 and superior S1 endplate osteotomies. Second-stage: Vascular-assisted retroperitoneal approach at L4–S1 was undertaken protecting the great vessels. Completion of osteotomies at L4 and S1 to En-bloc L5: (L4 inferior endplate, L4/5 disc, L5 body, L5/S1 disc and S1 superior endplate). Anterior reconstruction used an expandable PEEK cage obviating the need for a third posterior stage. Reinforced with a patient-specific carbon plate L4–S1 promontory.

**Results:**

Patient rehabilitated well and was discharged after 42 days. Cyberknife of 30Gy in 5 fractions was delivered two months post-op. Despite left foot drop, she's walking independently 9 months post-op.

**Conclusion:**

These are challenging cases require a truly multi-disciplinary team approach. We share this technique for a dual stage TES and metal-free construct with post adjuvant SABR to achieve maximum local control in spinal oligometastatic disease. This case promotes our modified TES technique in the era of SABR and separation surgery in carefully selected cases.

## Introduction

Total En Bloc Spondylectomy (TES) is a radical surgical technique first pioneered by Katsuro Tomita for solitary spinal metastases (1, 2). Its use in oligometastatic lesions of the spine aims to improve prognosis and oncological curability for patients *via* complete surgical resection rather than the piecemeal excision.

## Case presentation

A middle-aged African female underwent previous excision of lipoma of the right shoulder in 2018. She then had recurrence of the lump and was diagnosed with pleomorphic liposarcoma of the right shoulder. This was treated with pre-operative neo-adjuvant radiotherapy and *en bloc* resection in 2018. She was otherwise fit and well, working full time and regularly active and independent of all activities of daily living.

She reported low back pain with left leg radiculopathy radiating to the lateral foot in April 2020 with no bladder or bowel disturbance. Her oncology team organised a computed tomography scan which showed a solitary L5 lytic bone metastasis. After referral to our specialist unit, we obtained a single-photon emission computed tomography to confirm this was a solitary oligometastatic lesion. She was not eligible for SABR due to the fractured posterior wall and the lesion abutting the thecal sack. The lesion was already known to be radio insensitive. Piecemeal, palliative decompression is generally not advisable for sarcoma due to the risk of aggravation and further seeding.

**Figure 1:**
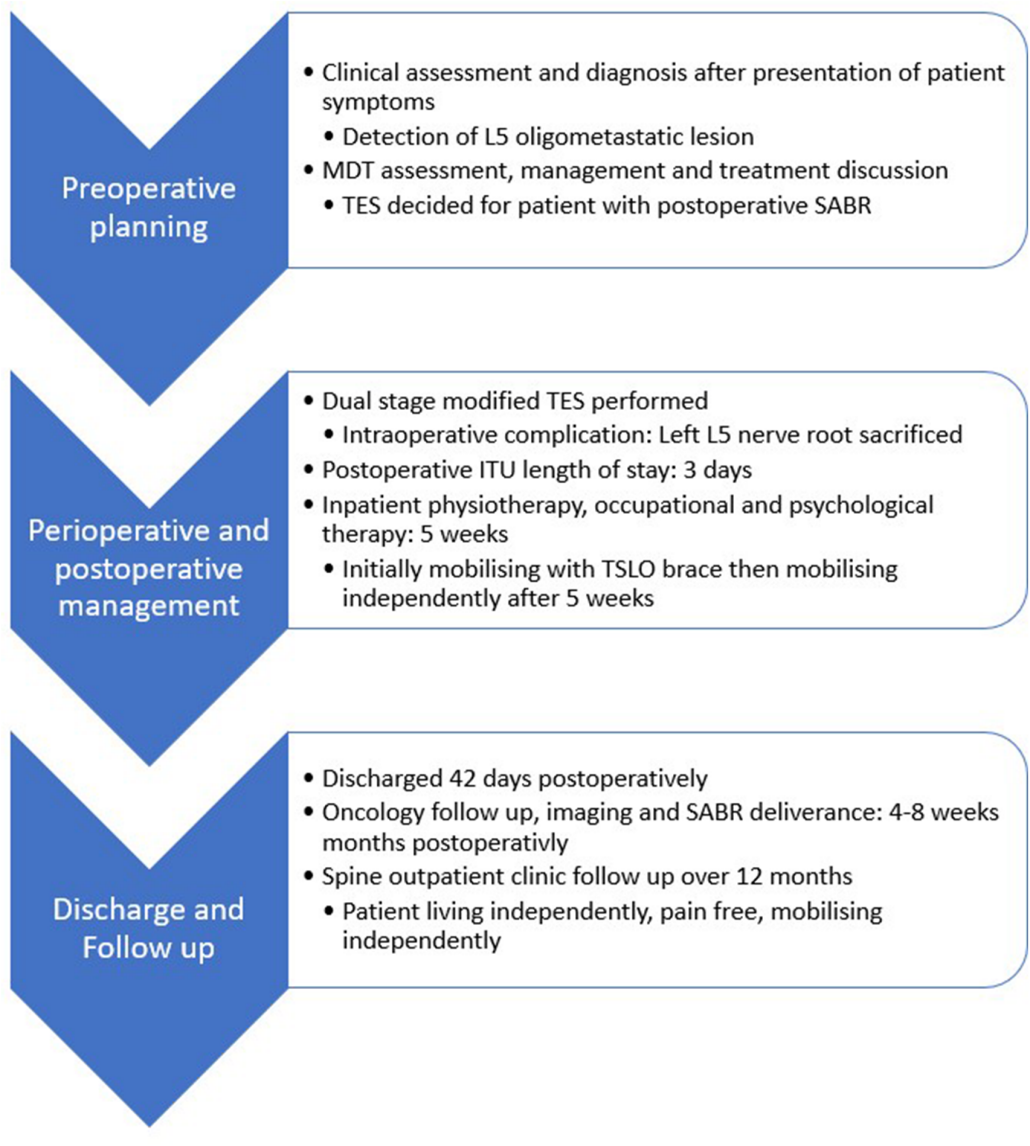
Patient Care Timeline.

She was referred to our complex Spine Multidisciplinary Team (MDT) meeting. The case was discussed between the oncologist, radio-oncologist and spinal team. The management plan was decided to be a L5 Tomita En bloc spondylectomy resection to provide maximal local control (Figure 1).

## Patient care timeline

### Pre-operative planning

The patient underwent Magnetic Resonance Imaging (MRI) whole spine, computerized tomography (CT) thorax, abdomen and pelvis, CT angiogram spine and whole-body Positron Emission Topography scan (PET). She underwent extensive pre-operative planning and was counselled on the risks of the surgery including high rate of morbidity and mortality. Consent for these procedures in view of Montgomery in the UK means that paternalistic medicine is not appropriate, and all options and risks must be advised to the patient. Particular note was given to permanent neurological deficit with L5 nerve root sacrifice but also injury to cauda equina. Vascular injury was of significant risk, and she was reviewed by the Vascular surgeon separately.

Carbon Fibre pedicle screws and rods were planned for this lady to ensure post-operative surveillance imaging could be optimal with no artefact. Furthermore, post adjuvant radiotherapy could be planned and delivered with more precision.

Carbon rods are rigid, and the pedicle screws have only 10 degrees of polyaxial movement making the insertion of this instrumentation technically challenging with little room for error. One method we employed to mitigate error was the use of 3-Dimensional printed model of the spine with bespoke jigs to allow pre-planned drilling of pedicles and insertion of carbon screws in a predetermined alignment.

En bloc resection of the posterior elements requires the pedicles to be osteotomised. Prior to this step, radiofrequency ablation of the pedicles was planned to help mitigate any living sarcoma tissue seeding.

A custom-built carbon spondylectomy cage was considered against an expandable poly-ether-ether-ketone (PEEK) cage. Fortuitously her pelvic incidence was 50 degrees and the L4–S1 lordosis of the PEEK Cage has an adaptable maximum lordosis of 25 degrees. A greater lordosis would have required a custom-made carbon cage.

Endoscopic equipment was prepared to help separate tumour from thecal sac as well as microscopic techniques to identify, isolate and preserve nerve roots but also to surgically sacrifice the right L5 nerve.

A multi-disciplinary team approach was required due to the complexity of the lesion and proximity to neurovascular structures. Teams involved included: Orthopaedic Complex Spine team, Neurosurgery, Vascular surgery, Anaesthetics, Radiologists, Intensive care, Physiotherapy and Occupational therapy.

### Surgical methods

The patient underwent a planned two-stage procedure with the primary aim being excision of L5 vertebra with stabilisation. She was categorised as an ASA 2 by the anaesthetic team. Spinal cord monitoring was undertaken for the duration of the operation.

First stage of the L5 Tomita en-bloc spondylectomy involved a posterior approach with dissection performed from L2 to pelvis. Skeletisation of the spine was performed, and the 3D printed navigation jigs were applied for segmental pedicle screw fixation with carbon fibre instrumentation. Carbon fibre pedicle screws were inserted from L2 to S2AI.

Intraoperative CT was performed to navigate the *en bloc* resection of the posterior elements of L5 requiring full removal of L4 and S1 posterior elements to fully visualise L5. Radiofrequency ablation was performed to the L5 pedicles with use of radiofrequency probes. The pedicles of L5 were cut with a navigated ultrasonic bone cutter allowing for precise cuts and protection of the nerve roots. Soft tissue dissection around the whole L5 posterior segment was performed.

Intraoperative microscope was utilised to perform careful separation of tumour from dura whilst preserving the tumour capsule and to safely mobilise nerve roots of L4–S1 and dissect surrounding soft tissue. An endoscopic set was at hand to assist in the separation of tumour from thecal sac. Epidural vessel bleeding was appropriately controlled.

A bovine dural patch was laid in front of the dura and anterior the thecal sac and to all nerve roots. Under navigation the ultrasonic bone cutter was then used to make posterior to anterior cuts through the endplate of L4 and S1. The osteotomy gaps were filled with haemostat and sealed with bone wax. The posterior neural arch of L5 was removed *en bloc*.

Calibration of the ultrasonic bone cutter is possible with the 3D CT and spinal navigation set. After the soft tissue separation of tumour from thecal sac and the L5 nerve roots were freed, the Carbon Fibre Rods were inserted, and we used cross connectors for added stability. Even with the 3D printed navigation jigs inserting screws for pre-planned alignment for a rigid rod, there was still significant technical difficulties creating the posterior construct.

Antibiotic-impregnated beads were then laid into the wound. Closure was performed in layers with a drain inserted.

The first procedure was approximately 15 h and involved both Complex Spine and Neurosurgical teams operating. The operation was undertaken successfully, and the patient remained stable throughout. Spinal cord monitoring remained satisfactory throughout the operation. She was kept in ICU overnight in preparation for the second stage the next day.

The second stage commenced with an anterior approach (retroperitoneal) performed with the Vascular team. A long crescent shaped anterolateral oblique approach was chosen coming from the left side. The bowel and great vessels were mobilised, and careful dissection was performed to expose the anterior aspect of the lumbar spine from L3 to S1. The vasculature to L5 was identified and smaller contributing vessels such as the iliolumbar veins were ligated. Sympathetic chain was visualised and protected when possible.

The previous L4 and S1 osteotomy cuts were identified and completed with use of the ultrasonic bone cutter again by cutting from anterior to posterior to meet the former cuts under direct vision and image intensifier. The L5 vertebra was removed and sent for histological analysis.

During the removal of L5 En bloc, a tear was caused to the left common iliac vein. This was repaired and the patient transfused intra-operatively. The removal of the L5 vertebrae also avulsed the left L5 nerve root taking the proximal root from within the thecal sac, a complication that was outlined as a strong possibility during the consenting process.

The expandable PEEK cage was assembled and filled with peptide enhanced bone graft. It was inserted under II guidance and expanded until press fit tightness and stability was achieved. The benefits of locking the posterior screws were expanding against a fixed point to deliver stability.

An overlying custom-made carbon plate and titanium screw construct was inserted covering L4 to S1. Cement augmentation was also applied for additional stability at two of the small screw sites holding the plate.

Closure was performed in layers and no further drains were inserted. Spinal cord monitoring showed some partial loss of left L5 nerve root, but activity was still noted due to cross over.

The second stage lasted approximately 9 h, with the total operating time over the two staged days equaling over 24 h. The patient was haemodynamically stable at the end of the surgery and was transferred directly to ICU.

### Post-operative management

The patient remained in Intensive Care Unit (ICU) and was transferred to the ward once stable a few days later. The patient made a steady recovery and had satisfactory post-operative check imaging (CT spine, MRI spine) with stable fixation noted.

She was reviewed daily by the Orthopaedic Complex Spine team with no major complications noted. Her left partial foot drop made small improvements during the time of her inpatient stay and she was fitted with an Ankle and Foot Orthosis splint for mobilising. She was able to mobilise as tolerated with the aid of a Thoracolumbar Sacral Orthosis brace. She received daily physiotherapy and occupational therapy and made good progress and was able to independently mobilise with the aid of a frame after 5 weeks.

She also received input from the pain team and cancer psychology support team during her admission. She developed no surgical site infections or post-operative complications such as chest infection, deep vein thromobis or pulmonary embolism.

A PET scan performed shortly prior to discharge revealed a right sided sacral fracture, although the patient was not symptomatic and did not affect her mobility. The scan also revealed the presence of possible metastases in the scapula and femur, and she was to be discussed at the Oncology MDT on discharge.

Patient was discharged after a total of 42 days. She continued her oncology follow-up which included Cyberknife deliverance of 30Gy in 5 fractions in accordance with UK consensus. The patient was well and mobilized independently pain-free 9 months postoperatively in spinal outpatient clinic with no local recurrence shown in PET scan.

## Discussion

The concept of “oligometastases” as an intermediate state between localised disease and widespread metastases was first proposed in 1995 by Hellman and Weichselbaum (3). The clinical implications of this are an opportunity to perform targeted local treatment of limited metastatic disease with the aim of potential curative treatment and progression-free survival. The spine is a common site of metastases and is a source of significant morbidity and mortality (4–6).

Tomita et al. developed the technique of Total En bloc spondylectomy (TES) *via* a two-step technique: an *en bloc* laminectomy *via* a posterior approach followed by *en bloc* resection of the anterior portion (vertebral body) with an oncological wide margin and subsequent insertion of vertebral prosthesis (1). Prior to this, conventional treatment involved piecemeal excision of malignant tissue which had a high possibility of tumour cell contamination of surrounding tissues, potentially contributing to incomplete tumour resection and recurrence of disease.

Previous reports of total corpectomy or spondylectomy for reducing local recurrence of a vertebral tumour showed positive clinical results (7–14). TES differs by involving En Bloc removal of the lesion *via* removal of the whole vertebra (both body and lamina) as one compartment (13).

Initial results for TES in thoracolumbar spinal metastases showed improved clinical outcomes such as pain relief, improved neurological deficit and prevention of impending paralysis (1). Longer-term follow up for patients undergoing TES showed mean length of survival was 38 months (rage 6–84 months) and 93% achieving local control and 32% still alive at last follow-up review (15). Similar encouraging prognostic outcomes have been shown with mortality rates less than 1%, morbidity less than 10% and median survival time longer than 3 years (16, 17).

### Stereotactic ablative radiotherapy

In relation to sarcoma, United Kingdom guidelines advise surgery as the gold standard for all adults with localised soft tissue sarcomas (18). The primary aim of surgery is to completely excise the tumour with a margin of normal tissue. Pre and/or post-operative radiotherapy is recommended along with surgical resection for majority of patients. Pre-operative treatment with chemotherapy and/or radiotherapy should also be considered depending on histology (18). Radiotherapy for intermediate and high grade sarcomas may be highly challenging, depending on the complexity of the affected body site, which could recommend the use of advanced stereotactic techniques (19).

Radiotherapy treatments for spinal oligometastatic disease include Stereotactic ablative radiotherapy (SABR). This method is beneficial as a precise high dose of radiation is targeted to the spinal lesions and causes tumour ablation whilst minimising damage to local healthy tissue (18). Several studies have shown the benefit of this treatment in oligometastatic spinal disease in outcomes such as tumour control, pain control, toxicity and morbidity (20–28).

CyberKnife is a non-surgical and non-invasive form of SABR that delivers effective tumour control (29, 30). In our patient, she was not eligible for this treatment due to the fractured posterior wall and the lesion abutting the thecal sack. In addition, the lesion was already known to be radio insensitive. Our MDT opted for surgical intervention in the form of L5 *en bloc* spondylectomy as piecemeal, palliative decompression is generally not advisable for sarcoma due to the risk of aggravation and further seeding. The patient underwent CyberKnife SABR post-operatively as part of her ongoing oncological management.

### Carbon fibre constructs

Carbon fibre implants have increasingly been used because metal hardware can limit post-operative radiotherapy due to its scattering effect of ionising radiation. Carbon fibre fixation systems (including rods and screws) can make post-operative radiotherapy easier and more effective due to its radiolucent nature and reduced interference with ionising radiation and accelerated particles (30). Studies have shown the benefit this intervention on improving radiotherapy treatment accuracy and its radiolucent benefit in the follow-up of patients to allow early detection of local recurrence (31–33). For these reasons, we decided to utilise carbon fibre pedicle screws and rods in combination with an expandable PEEK cage. To help protect the cage from migrating, we applied a custom carbon plate anteriorly.

Titanium within the target area introduces imaging artefact to the planning CT due to its very high electron density causing beam hardening, partial voluming and missing projection data, making it harder to visualize and accurately delineate the target for treatment. An MRI scan is used to help delineate the target and organs at risk, including the spinal cord / cauda equina; this scan is also affected by metal artefact which not only reduces its usefulness for delineation but also makes the task of registering the MRI to the planning CT much more difficult. Metal artefact on CT misrepresents the electron density in the area surrounding the metal, leading to inaccuracy in the calculation of dose in these regions. Furthermore, the dose calculation algorithms used by treatment planning systems are known to be less accurate at boundaries between tissues of different densities, under-estimating the dose at the interface between tissue and metal (caused by backscatter) and over-estimating the dose in the shadow of the metal (caused by increased attenuation) (34). When planning pelvic SABR treatment for patients with prosthetic hips, planning strategy would be to avoid allowing beams to enter through the prosthesis; however, this strategy is not practical for vertebral SABR, where the metalwork immediately surrounds the target area and may even pass through it.

**Figure 2:**
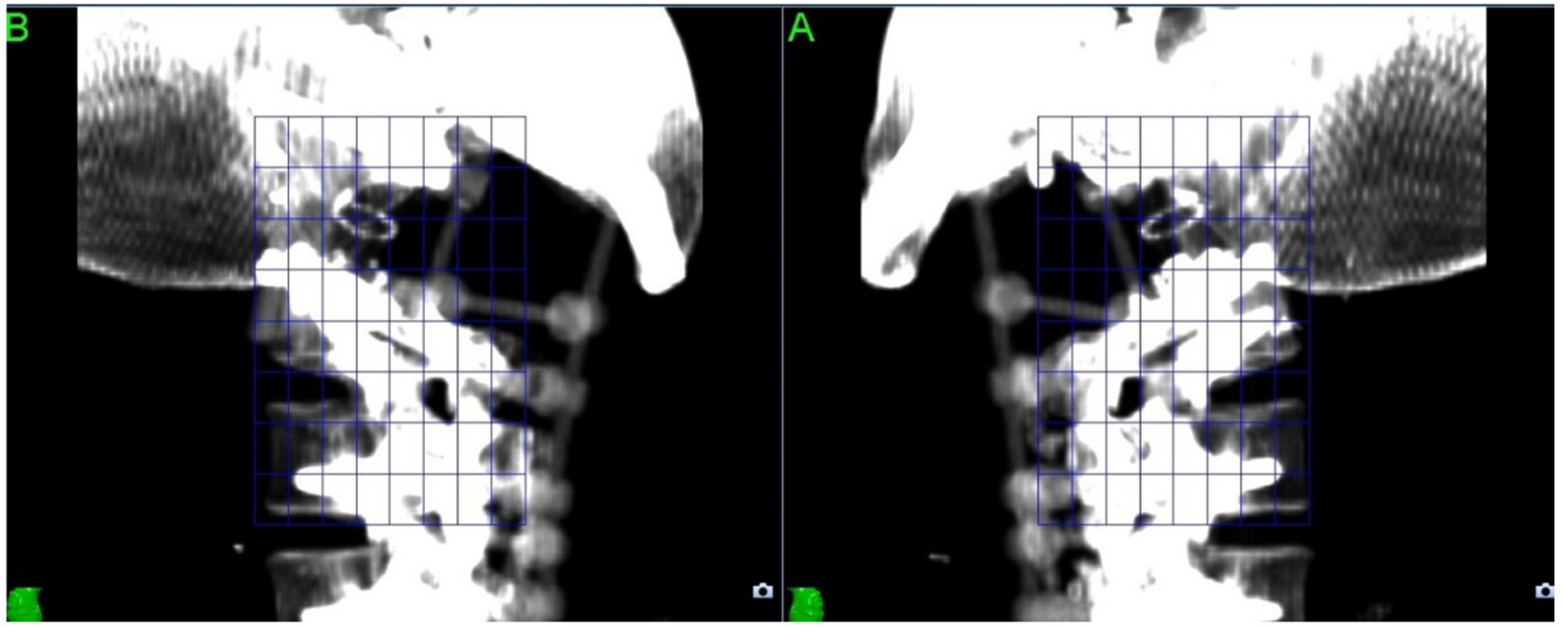
X-Sight Spine tracking DRRs from planning system.

For CyberKnife SABR treatments, the imaging artefact can also cause issues with the X-Sight Spine tracking method used to track the position of the target throughout treatment. (Figure 2) The tracking method uses a feature-based recognition algorithm to identify the patient position from kilovoltage images acquired every 45–90 s; this cannot be performed reliably if the images are compromised by metal artefact, so the tracking “mesh” has to be placed further away from the target.

Using carbon rather than titanium for the reconstruction resolves all these issues as the density of carbon (1.8 g/cm^3^) is much lower than that of titanium (4.5 g/cm^3^), so does not cause artefact in the planning image and can be modelled more accurately by the planning system.

### Cyberknife dosage fractionation

**Figure 3:**
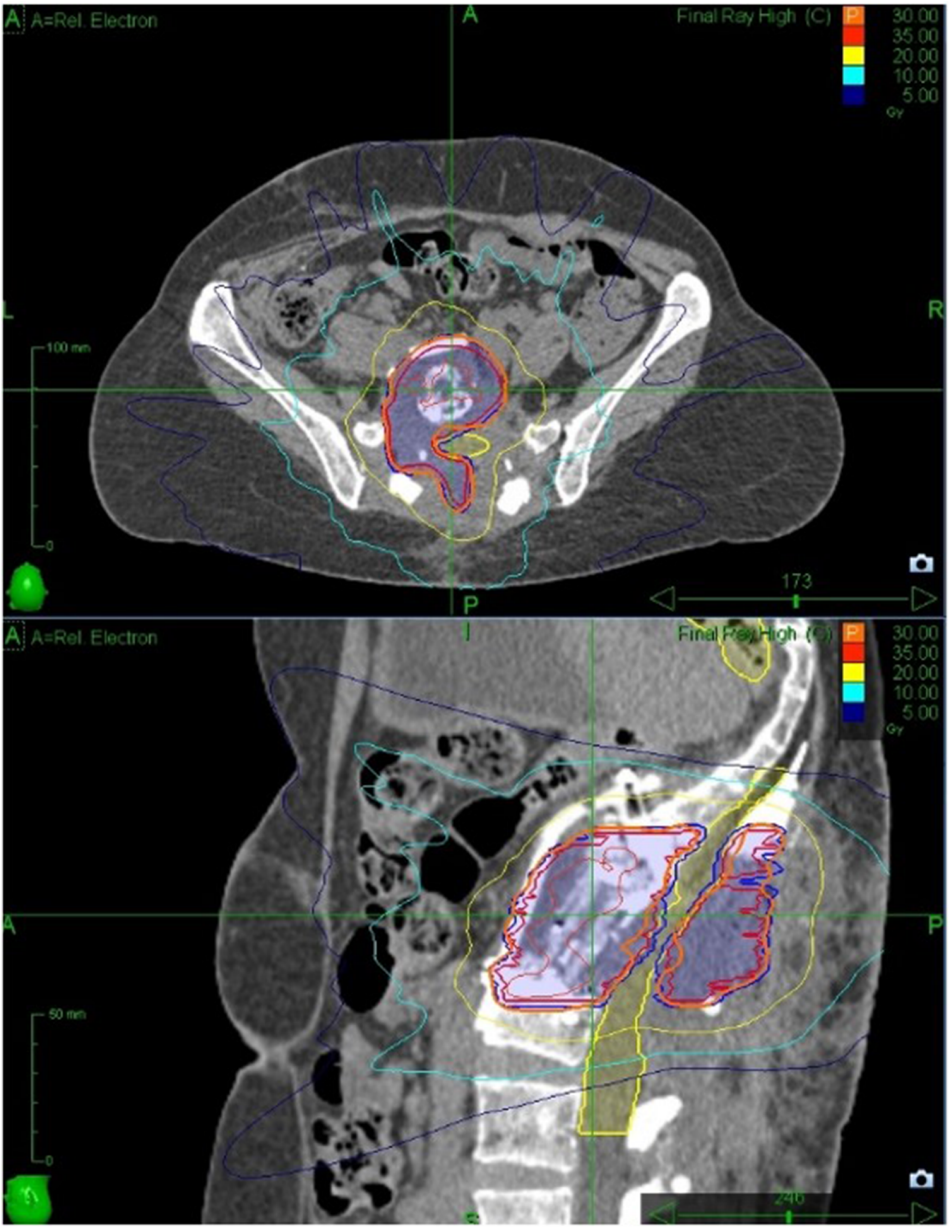
Dose distribution from planning system.

The Gross Tumour Volume and Clinical Target Volume were drawn following consensus guidelines (35) and expanded by 2 mm for PTV according to local protocol. Prescribed dose was 30Gy delivered in 5 fractions in order to meet the UK consensus dose constraint for bowel (36), with Planning Target Volume coverage compromised to meet the cauda equina (2 mm Planning Organ as Risk Volume) dose constraint. Treatment planning was performed using Accuray Precision version 2.0.1.1 and treatment was delivered using a CyberKnife VSI with the Iris collimator. The treatment plan comprised 240 non-coplanar, non-isocentric beams with an estimated delivery time of 45 min per fraction. Because of the use of carbon fixtures for the reconstruction, we were able to track directly on the target area with no issues (Figure 3).

### Custom 3d printed surgical navigation guide

To aid with the carbon fibre pedicle screw placement, we used a custom 3D printed surgical navigation guide (37). Custom 3D printed guides have been shown in a recent systematic review and meta-analysis to reduce operative time, blood loss and achieve excellent screw placement compared with freehand techniques (38). We specifically required the guide to enable the S1 screws divergence to allow for the S1 cuts. The S2AI screw head alignment needed to be perfectly aligned with lumbar segment as there is no flexibility and is totally rigid with the construct. The low polyaxial nature of the screws makes them similar to monoblock screws which means cephalad caudal alignment is as important to medial lateral alignment so that the rod sits flush to the tulip at each level.

### Radiofrequency ablation

Prior to pedicle insertion, we used cool Radiofrequency Ablation System for theoretical destruction of sarcoma cells to prevent live tissue seeding. Studies have also shown its benefit in pain improvement in patients with metastatic bone disease (39).

### Spinal surgery infection prophylaxis

Following fixation of the cage construct, we applied antibiotic-impregnated calcium sulphate beads into the wound upon closure. These beads have been increasingly used in certain orthopaedic procedures as it has proven efficacy against biofilms and has predictable supra-therapeutic antibiotic elution profile over 40 days (40, 41). It has also been shown to be beneficial in several spinal implant fixation cases (42–44).

### Peptide enhanced bone graft

The expandable PEEK cage was filled a peptide enhanced bone graft which allows ectopic bone growth on the implant only. Studies have shown its high efficacy in spinal fusion rates with good post-surgical outcomes, including in patients with poor bone regenerative capacity quality (45, 46).

### Multidisciplinary approach

As this was a surgically complex case with high risk for morbidity and mortality, we adopted a multi-surgical specialty approach including the help of our hospital Vascular surgery team. The anterior approach to the lumbar spine is often not favoured by spinal surgeons or neurosurgeons due to the unfamiliarity and potential risk of serious vascular or visceral damage (47). Vascular complications are often related to the need to mobilise the great retroperitoneal vessels and other adjacent structures for exposure to the anterior lumbar spine. It is argued that the operating team should require vascular and general surgical skills in order to both perform the exposure and deal with any resulting complications (47). Although there has been debate about whether the presence of an “access surgeon” has a beneficial effect on complication rates for anterior lumbar spinal surgery, a recent systematic review and meta-analysis did report lower overall postoperative complication rates, lower reoperation rates and lower prosthesis complications and recommended availability of an access surgeon where exposure may be difficult (47–50). We greatly appreciated the expertise of our vascular surgeon and indeed required his specialist input intra-operatively when there was a tear to the left common iliac vein which was successfully repaired.

## Take-away lessons

We present a challenging case of an L5 *en bloc* spondylectomy for a case of oligometastatic liposarcoma performed at our specialist complex spinal unit. Our reported extensive pre-operative planning and specialist intra-operative techniques may be of assistance to others taking on these surgically challenging cases. We recommend a truly multi-disciplinary team approach for pre, intra and post-operative stages when managing such complex cases including Complex Spine team, Neurosurgery, Vascular surgery, Anaesthetics, Intensive care, Radiologists, Radiotherapy Physicists, Oncologists, Physiotherapy, Occupational therapists, Psychologists and specialist nursing staff.

## Patient perspective

Thank you very much to the spinal team and my doctors that performed this surgery and saving my life. I received excellent care before I got admitted, before my operation, during my hospital stay and was kindly looked followed up when I went home. The surgeons were very reassuring and provided me with good care. I also received lots of reassurance and support during my cancer hospital follow up when getting radiotherapy after my operation.

## Data Availability

The raw data supporting the conclusions of this article will be made available by the authors, without undue reservation.
